# Supplementary material for: Ki67 as a Predictor of Response to PARP Inhibitors in Platinum Sensitive BRCA Wild Type Ovarian Cancer: The MITO 37 Retrospective Study
Source: Cancers (Basel). 2023 Feb 6;15(4):1032. doi: 10.3390/cancers15041032 (PMC9954459; doi:10.3390/cancers15041032)
Supplement: Supplementary file 1 [file cancers-15-01032-s001.zip › cancers-2211475-supplementary.pdf]

Supplementary Materials:

Table S1: Clinical sites participating to MITO 37 study.

Centers		No. of included patients
1	Ospedale Mauriziano di Torino, Oncologia ginecologica;	7
2	Istituto Romagnolo per lo studio dei Tumori 'Dino Amadori';	2
3	IRCCS Candiolo;	18
4	Ospedale Sant'Anna di Torino;	22
5	Istituto Nazionale Tumori di Milano;	20
6	IRCCS San Martino IST, Genova;	11
7	Fondazione IRCCS Policlinico San Matteo di Pavia;	11
8	Ospedale di Parma, Oncologia Medica;	8
9	IRCCS Reggio Emilia;	4
10	Istituto Oncologico Giovanni Paolo II, Bari;	3
11	Ginecologia ed Ostetricia, Policlinico, Bari;	7
12	Department of Life, Health & Environmental Sciences, Università dell'Aquila;	6
13	Dipartimento di Oncologia, Ospedale di Udine;	16
14	Ulls 2 Marca Trevigiana Ospedale Ca' Foncello Treviso;	12
15	Unità di Ostetricia e Ginecologia, Dipartimento di Biomedical and Human Oncological Science (DIMO), Università di Bari	3
